# Notes and Comments

**Published:** 1899-03

**Authors:** 


### Notes and Comments.[1]

THE NON-REMOVAL OF SOFTENED DENTINE BEFORE FILLING.—Dr. J. Leon Williams describes his treatment in the *Items of Interest*, about as follows: This is a question that can hardly be answered offhand, or without the use of several ifs and buts. *If* there has been no inflammation or but slight inflammation of the pulp, I think it is perfectly safe to leave a layer of partially softened dentine over the pulp, *providing the proper treatment of this softened dentine has been carried out.* And this is my idea of the proper treatment in such cases.

Remove the softer portions of dentine and place a pledget of cotton-wool saturated with absolute alcohol in the cavity. Leave this for one minute, then remove, dry the cavity, and flood it with oil of cloves, which also leave for one minute. Any one accustomed to histological work will see the *rationale* of this treatment at a glance. Oil of cloves, which is known to the histologist as one of the most powerful *clearing* agents known,—*i.e.*, it has the property of very rapidly penetrating any tissue, even bone and dentine, that has previously been treated with strong alcohol. It is a sufficiently good germicide for the purpose, and it seems also to have a medicinal effect of value in slight congestion of the pulp. Used as above described, it will penetrate a considerable thickness of dentine and thus search out and destroy or render inert any forms of bacteria that may have penetrated beyond the point where you have cut. Dry out the excess of oil of cloves and varnish the bottom of the cavity with Canada balsam, dissolved in chloroform, to which has been added ten per cent. of the solution of hydronaphthol in chloroform previously spoken of. For this use, the balsam is dissolved in chloroform instead of turpentine, because here we wish it to dry rapidly, while in the treatment of the root-canal we do not wish it to dry rapidly. Partially dry the layer of varnish in the bottom of the cavity with hot air and then apply to the floor of the cavity a piece of thick asbestos paper cut the proper size

[1] The assistant editor solicits contributions for this department,—new methods, new remedies and formulas, or any short practical note which may prove of value to the practitioner or student. Address 1338 Walnut Street, Philadelphia.

and shape. The partially dried varnish will hold the asbestos paper firmly in place. Now line the cavity with quick-setting cement and fill with gold or amalgam. Such treatment will leave the tooth reasonably free from sensitiveness to thermal change even when the pulp is nearly exposed.

Clacking of full Dentures.—To prevent the annoying clacking or rattling of full dentures, Dr. F. Mackinzie recommends, when constructing them of vulcanite, the removal of the four molar blocks before packing, and packing in their place white vulcanizable rubber, so that there will be four pads of softer material than the teeth, which, when they come into contact, will reduce the rattle.

A. M. Didier, of Paris, about half a century ago, to remedy the same annoyance in dentures of mineral paste (closely resembling continuous gum) made holes or cavities in the crowns of the molar teeth, and filled them with wood or ivory.

Artificial Nose of Celluloid.—Herr Walther Bruck, of Breslau, suggests the following procedure for constructing an artificial nose. Upon a model of the remains of the nose and the surrounding parts a suitable nose is carved, and duplicated in zinc or lead. This is first painted with a solution of gum-arabic, and then a layer of damp kid leather is smoothly laid on. A piece of white transparent celluloid, one half millimetre in thickness, is softened in acetone (acetic ether), and carefully pressed into shape on the metallic cast so prepared. When the celluloid has hardened, it is removed, and any slight changes in form corrected by pressing it again upon the slightly warmed metallic cast. When cold, the celluloid is trimmed to shape, its edges smoothed, and the surface gloss removed by rubbing with powdered pumice-stone. Suitable retaining fixtures are attached by means of celluloid dissolved in acetone. It is then painted on the under surface to match the complexion of the face. Herr Bruck claims that the color seen through the celluloid produces a more natural appearance.—*Dental Record*, London, October 1898, p. 463.

Use of Tin-Foil in Model and Bite Impressions.—Now and again, when taking impressions for regulating cases, crown- and bridge-work, or for small partial plates, etc., it is more convenient, and greater accurracy is attained, by allowing the patient to bite into the impression so as to secure at the same time and in the same mass of impression material a mould for the model and for the occluding teeth. Time and effort is saved, and the risk of error in adjusting a separate impression of the occluding teeth to the model is avoided. It has the disadvantage, however, when, as is frequently the case, the upper and lower teeth meet in actual contact, that there may not be enough substance of impression material to hold that which is outside the teeth to that which is within, or, the two impressions may so completely coalesce that it is difficult, and at times impossible after they are filled with plaster to separate the two casts so as to preserve accuracy at these points. This may be effectively overcome by inserting in the impression material before placing it in the mouth, at the point about where the teeth will meet, a sheet of thick tin-foil, or "tea lead," or the thin metal such as is used to cover the face of models used in vulcanite work. This, while quite efficient in separating the impressions of the upper and the lower teeth, is sufficiently thin and pliable not to affect their accuracy. In some cases it may be more convenient to place a mass of impression material upon the upper and lower teeth, lay the tin-foil upon either one, and direct the patient to close the jaws firmly. For crown- and bridge-work, it is convenient to keep on hand blocks of impression material of suitable size, made of two layers separated by a sheet of the foil.

---

LIQUID GLUE.—Allow the glue to swell in water for ten hours, then melt in a water bath and add one-tenth its weight of salicylate of soda.

